# Comparative analysis of muscle coordination patterns underlying different types of stepping movements

**DOI:** 10.1007/s00221-025-07118-4

**Published:** 2025-07-08

**Authors:** Lotte Hagedoorn, Edwin van Asseldonk, Vivian Weerdesteyn, the HEROES Consortium, Aurora Ruiz Rodríguez, Ilse Leijen, Erwin van Wegen, Gert Kwakkel, Ilona de Rooij, Noël Keijsers, Maarten Prins, Mariska Janssen, Marissa Riemens, Juha Hijmans

**Affiliations:** 1 Department of Rehabilitation, Donders Institute for Brain, Cognition and Behavior, Radboud University Medical Center, Nijmegen, The Netherlands; 2 Faculty of Engineering Technology, University of Twente, Enschede, The Netherlands; 3 Sint Maartenskliniek Research, Nijmegen, The Netherlands

**Keywords:** Muscle synergies, Neuromuscular control, Electromyography (EMG), Reactive stepping, Voluntary stepping, Balance recovery.

## Abstract

Reactive stepping is crucial for preventing falls after losing balance. While perturbation-based training improves reactive step quality, voluntary step training appears less effective. To gain insight into the neural underpinnings of such task-specific effects, we examined the muscle coordination patterns of voluntary and reactive stepping. As an additional step type, we introduced action observation with motor simulation of reactive steps, as it has shown promise for improving reactive step quality without requiring real balance perturbations. Electromyographic signals were recorded from eight leg and trunk muscles of healthy young subjects (*n* = 15) during three step types: (1) reactive stepping following support-surface translations, (2) voluntary stepping in response to a visual stimulus, and (3) action observation with motor simulation of reactive steps, as demonstrated by a human actor. Each condition involved stepping with the right leg in five directions (anterior/45°anterior/lateral/45°posterior/posterior). Muscle synergy analysis was employed to identify muscle weights with corresponding temporal activation profiles, which were compared across step types. Step characteristics and body configurations at foot down were also compared. Three muscle synergies were consistently recruited across participants and step types. In reactive stepping, a majority of participants exhibited a fourth muscle synergy involving rectus femoris and soleus. Temporal activation coefficients and body configurations varied with step type. While largely similar muscle weights were found for the three types of stepping movements, higher levels of activation in reactive stepping presumably reflect the greater biomechanical challenge involved. These findings may help explain differences in effects between different step training protocols.

## Introduction

The risk of falling is increased in older adults and people with neurological disorders such as stroke (Ambrose et al. 2013; Lewis and Griffin 2023; Weerdesteyn et al. 2008). Adequate stepping responses are essential to prevent falls in daily life situations (Maki and McIlroy 2006). In particular, reactive stepping provides a final common saving strategy following a loss of balance, with poor reactive stepping being associated with a greater risk of falling (Okubo et al. 2021). To improve reactive stepping performance, perturbation-based balance training (PBT; e.g. by responding to sudden support-surface movements), has shown to be effective in various populations (Mansfield et al. 2015; McCrum et al. 2022). Voluntary step training is another commonly used method, however, recent studies demonstrated that PBT is superior in improving reactive balance capacity, highlighting the importance of task-specific training (Grabiner et al. 2014; Kim et al. 2021). Indeed, voluntary steps are different in nature as they typically occur in anticipation to a potential balance disturbing hazard in order to avoid a loss of balance (Okubo et al. 2021).

We used muscle synergy analyses to study the muscle coordination patterns in the stepping leg to gain further insight into the differences between voluntary and reactive stepping at the level of neuromuscular control. Muscle synergy analysis is an established method for assessing muscle recruitment patterns (Staring et al. 2024; Ting and Chvatal 2010; Torres-Oviedo and Ting 2007). Muscle synergies are represented as muscle weights, organized as time-independent spatial coefficients, with corresponding temporal activation coefficients. This approach is particularly useful for understanding impaired muscle activity coordination across various neurological disorders. Previous studies comparing perturbed and unperturbed walking showed largely similar muscle weights, while temporal activation coefficients and movement kinematics differed (Brüll et al. 2024; Chvatal and Ting 2012; Oliveira et al. 2012). Yet, a recent study that compared muscle synergies between voluntary and reactive stepping from quiet stance found a low similarity between reactive and voluntary steps (Wang et al. 2021). Fewer muscle synergies were used during reactive stepping than during voluntary stepping, possibly due to co-activation of more muscles with less complexity during reactive stepping. Yet, as this study included only a single backward reactive stepping trial for each participant, a more comprehensive evaluation is warranted for drawing conclusions on potential differential neuromuscular control.

To provide further insight into the muscle coordination patterns underlying the different types of stepping, we included multiple stepping directions with repeated trials in the present study. This is considered critical for having sufficient meaningful variance to allow conducting a sound muscle synergy analysis (Ting and Chvatal 2010). Our focus in the present study is on the execution of the step itself, as prior to lifting the foot, voluntary and reactive step initiation inherently differ due to the recruitment of anticipatory postural adjustments or automatic postural responses, respectively (Santos et al. 2010). In addition, we introduced action observation with motor simulation of reactive stepping as an intermediate type of stepping, as it has shown potential for improving reactive stepping responses without requiring real balance disturbances (Hagedoorn et al. 2024, 2025). Action observation with motor simulation of reactive stepping, as defined in this work, involves observing someone else recovering from a balance perturbation with a reactive step, while imagining experiencing the loss of balance oneself, and physically simulating the reactive step according to the given example. As mental imagery of reactive stepping has been demonstrated to activate cortical areas similar to the actual performance of reactive steps (Bhatt et al. 2018), we expected action observation with motor simulation of reactive stepping to more closely resemble the neuromuscular control of actual reactive stepping (as opposed to voluntary stepping) task. We implemented muscle synergy analysis to compare muscle weights and their corresponding temporal activation coefficients (Torres-Oviedo and Ting 2007). In addition, we compared spatiotemporal step characteristics and body configurations between step types. We expected to find differences between reactive and voluntary stepping, with intermediate results for action observation with motor simulation of reactive stepping.

## Methods

### Participants

We included fifteen healthy young individuals (24 ± 3 years old, 11 females) in this study, similar to sample sizes of previous studies on muscle synergy analyses of posture and gait (Chvatal and Ting 2012; Oliveira et al. 2012; Wang et al. 2021). Exclusion criteria were any neurological, orthopedic or vestibular disorders or use of medication negatively affecting balance, or a body mass index above 25 kg/m^2^ to ensure sufficient quality of the electromyography (EMG) recordings. All subjects gave written informed consent prior to participation. The study was conducted in accordance with the declaration of Helsinki and adhered to the guidelines of the local medical ethical committee (CMO region Arnhem-Nijmegen).

### Experimental protocol

Participants completed three blocked step type conditions in a random order. Each block comprised 60 trials of either reactive, voluntary or action observation with motor simulation of reactive stepping, henceforth referred to as motor-simulated stepping. All steps were performed with the right leg, as prior studies revealed no substantial differences between stepping with the left and right leg (Staring et al. 2024; Wang et al. 2021). Each condition consisted of 12 steps in each of five directions: anterior (Ant), 45° anterior (AntLat), lateral (Lat), 45° posterior (PostLat) and posterior (Post). The stepping directions were then alternated in a random order within each condition. Participants received up to five familiarization trials before each blocked step type condition.

**Fig. 1:**
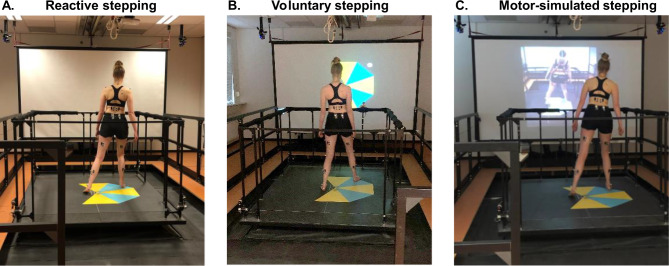
Experimental setup of (a) the reactive stepping condition, (b) the voluntary stepping condition and d) the motor-simulated stepping condition. Participants stood on a step mat that was placed on the Radboud Fall Simulator

Reactive steps were evoked by sudden balance perturbations (Fig. 1a). Balance perturbations were delivered by the Radboud Fall Simulator, which is a movable platform (Baat Medical, Enschede, The Netherlands) with two embedded force plates (0.6 m by 1.8 m each, AMTI Custom 6 axis composite force platform, USA). A reactive step in the target direction was elicited by a platform translation in the opposite direction (e.g., a backward reactive step was elicited by a forward platform translation). Perturbations comprised an acceleration (300 ms), constant velocity (500 ms) and a deceleration phase (300 ms), with a perturbation intensity of 2 m/s^2^. Inter-trial intervals randomly varied between 3 and 6 s. The participants were instructed to respond to the balance perturbations with a single step without grabbing the rails surrounding the platform.

Voluntary steps were performed in response to a visual stimulus shown on a large screen in front of the platform, on which the step mat was also displayed (Fig. 1b). The visual stimulus was a stationary white circle that appeared after a random period of 3 to 6 s. Participants were instructed to perform a single step as fast as possible in the direction indicated by the stimulus.

Motor-simulated steps were performed in response to video recordings of reactive steps demonstrated by an actor, which were shown on the screen (Fig. 1c). The video showed platform perturbations with randomly varying inter-trial intervals (3, 3.5, 4 s). Participants were instructed to attentively observe the actor’s reactive step, to imagine experiencing the loss of balance themselves and to step along with the actor as accurately as possible.

To ensure a comparable step size across all three step type conditions, we used a custom-designed vinyl step mat to indicate the target step size of 53 cm (i.e., corresponding to the actor’s step size) and target step directions using colored triangles in each condition. Participants stood on the step mat barefoot with their feet 4.5 cm apart. This narrow stance width forced participants to always perform a reactive step in the event of balance perturbations with a lateral component. Participants were instructed to land their foot approximately on the opposite edge of a triangle while continuing to look at the screen in front. We emphasized that accuracy of foot placement did not have to be prioritized. After having completed a step, participants returned to the starting position which was indicated on the step mat.

### Data acquisition and pre-processing

Ground reaction forces under each foot were collected by the two force plates integrated in the movable platform (sampled at 2000 Hz). The vertical force component from each force plate was filtered offline with a 20 Hz tenth-order zerophase Butterworth filter. The instants of foot off and foot down were detected from the ground reaction forces.

Reflective markers were placed on anatomical landmarks according to the Vicon Full Body Plugin-Gait model (Vicon Motion Systems, United Kingdom), excluding markers on the head and arms. An additional reflective marker was placed on the platform to correct marker positions for platform movements (i.e., in the reactive step condition only). Marker positions were recorded by an 8-camera 3D motion capture system (Vicon Motion Systems, United Kingdom) (100 Hz). Marker trajectory data were filtered offline with a 10 Hz second-order low-pass zero-lag Butterworth filter.

EMG signals were recorded from eight leg and trunk muscles using surface EMG electrodes (Mini Wave, Cometa Systems, Italy). These recordings were synchronized with the ground reaction forces and marker trajectories. The muscles included were the right erector spinae, gluteus medius, biceps femoris, rectus abdominis, rectus femoris, peroneus longus, tibialis anterior and soleus. EMG electrodes were placed according to SENIAM guidelines. Raw EMG signals were successively offline bandpass filtered (20–250 Hz), Hilbert transformed and lowpass filtered (40 Hz) (Staring et al. 2024). EMG signals were subsequently time-normalized from the instants of foot off (0%) to foot down (100%), resulting in a total 101 time samples. By concatenating the filtered EMG trials end to end, a distinct matrix ($\:\mathbf{V}$) was created for each participant and condition independently, with each row corresponding to a single muscle.

### Muscle synergies

We used non-negative matrix factorization (NNMF) to extract muscle synergies, a method commonly used for dimensionality reduction of EMG data (Ting and Chvatal 2010; Torres-Oviedo and Ting 2007). NNMF factorizes a set of measured EMG data ($\:\mathbf{V}$) into muscle weights ($\:\mathbf{W}$) and temporal activation coefficients ($\:\mathbf{H}$), such that reconstructed EMG patterns (i.e., $\:\mathbf{W}\cdot\:\mathbf{H}$) approximately equal the non-factorized data set $\:\mathbf{V}$ again. The muscle weights represent the relative contribution of the muscles, i.e., the time-independent spatial coefficients, and the temporal activation coefficients represent the activation of the muscle weights as function of the step cycle.

The lowest number of muscle synergies accounting for at least 80% of the variance (i.e., the variance accounted for (VAF)) was extracted, consistent with our previous study on muscle synergies (Staring et al. 2024). Muscle synergies were extracted for each condition (i.e., step type) and participant separately. Directions were not separated prior to muscle synergy extraction, as including multiple directions is considered critical to ensure sufficient variance for a sound muscle synergy analysis (Ting and Chvatal 2010). Before extracting muscle synergies, $\:\mathbf{V}$ was normalized to unit variance by dividing each row by its standard deviation to weigh each muscle equally in the factorization, which was undone after factorization to restore the original scaling. We then performed functional sorting to group muscle synergies based on similarities using an iterative process according to the procedures described by Ting et al. (Torres-Oviedo and Ting 2007). Muscle weights were considered similar when Pearson’s correlation coefficient ($\:r$) was statistically significant (i.e., $\:r\ge\:0.622$ for $\:\alpha\:=0.05$ for a one-tailed test and $\:df=\:8\:$muscles$\:\:-2=6$). This iterative process was repeated until ≥ 90% of all individual muscle synergies (of all directions and participants for the particular condition) were assigned to a group synergy. If an individual muscle synergy qualified for multiple group synergies (i.e., $\:r\ge\:0.622$), it was assigned to the group synergy for which the $\:r$-value was highest.

Finally, mean temporal activation coefficients were calculated for each group muscle synergy, condition and direction independently.

### Step characteristics and body configurations

We calculated the following outcome variables. Step length was used as a control variable and was calculated as the displacement of the mid-foot coordinate (i.e., mid-point between the calcaneus and the second metatarsal) in the step direction between perturbation or cue onset and the instant of foot down (Staring et al. 2024). Descriptive variables were step duration (the time interval between the instants of foot off and foot down), step velocity (step length divided by step duration), and leg angle and trunk angle at the instant of foot down (Roelofs et al. 2019). The leg angle was defined as the angle between the vertical and the line connecting the mid-pelvis to the second metatarsal of the stepping foot. Larger leg angles correspond to a mid-pelvis position more distant from the foot position in the transverse plane. The trunk angle was defined as the angle between the vertical and the line connecting the mid-shoulder to the mid-pelvis. Larger positive trunk angles represent a greater trunk inclination towards the step target, whereas negative trunk angles indicate a trunk inclination away from the step target. All outcome measures were calculated with custom-written Matlab software (version R2022a).

### Statistical analysis

A Friedman’s test was conducted to compare the number of muscles synergies accounting for ≥ 80% of the variance across step types. Fisher’s exact tests were performed to evaluate the presence of group synergies across step types. We employed linear mixed models to examine the within-synergy effects of *step type* and *time* on the temporal activation coefficients. The model included fixed effects for *step type*, *time*, and the interaction of *step type* and *time*. Random intercepts were included for each participant within each step direction, accounting for within-direction variability across participants. Because the activation coefficients are time-dependent, and interaction effects were significant, we conducted post-hoc comparisons per time sample with Bonferroni correction to adjust for multiple testing. Similarly, linear mixed models followed by posthoc pairwise comparisons with Bonferroni correction were used to examine the effects of *step type* and *step direction* on the step characteristics and body configurations, accounting for variability across participants (fixed effects were *step type*, *step direction* and the interaction of *step type* with *step direction*; random effects were specified for each participant within each step direction). Before employing the linear mixed models, Shapiro-Wilk tests indicated no violations of residual normality. Outcome values (trial average for each direction) were used in the linear mixed models. Statistical analyses were performed in SPSS (version 25) with a significance level of α = 0.05.

## Results

**Fig. 2:**
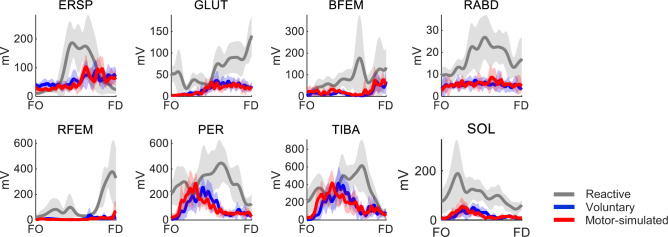
Examples of EMG activity during reactive, voluntary and motor-simulated stepping. The EMG activity of an individual subject in the sideward direction is shown from the instants of foot off (FO) to foot down (FD) as the mean (solid line) ± standard deviation (shaded area) of multiple trials. Erector spinae (ERSP), gluteus medius (GLUT), biceps femoris (BFEM), rectus abdominis (RABD), rectus femoris RFEM), peroneus longus (PER), tibialis anterior (TIBA) and soleus (SOL)

Participants successfully completed the stepping trials without falling into the harness. We included EMG data (Fig. 2) of an average of 52 ± 7 reactive steps, 55 ± 3 voluntary steps, and 53 ± 7 motor-simulated steps per participant out of 60 trials per condition. Trials were excluded from the muscle synergy analysis because of incorrect stepping direction or length (i.e., foot not touching the outer edge of the step mat), more than one step taken, or technical malfunctioning.

### Muscle synergies

**Fig. 3:**
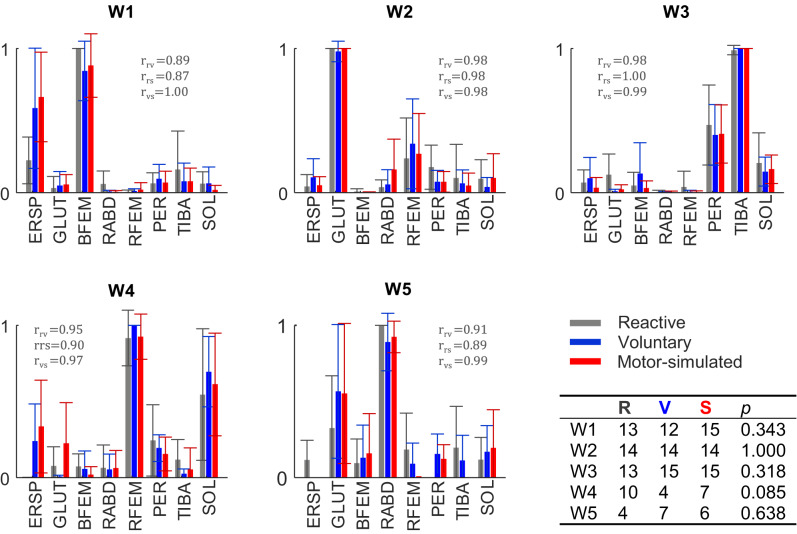
Muscle weights (W1-W5) per step condition. Each bar represents mean and standard deviation across subjects. Correlation coefficients values indicate the between-step type similarity of group synergies (reactive vs. voluntary ($\:{r}_{\text{r}\text{v}}$), reactive vs. motor-simulated ($\:{r}_{\text{r}\text{s}}$), voluntary vs. motor-simulated ($\:{r}_{\text{v}\text{s}}$). Erector spinae (ERSP), gluteus medius (GLUT), biceps femoris (BFEM), rectus abdominis (RABD), rectus femoris RFEM), peroneus longus (PER), tibialis anterior (TIBA) and soleus (SOL). The table shows the number of participants out of the total sample size of *n* = 15 in whom a muscle synergy was present, and the *p*-values of Fisher’s exact tests on the differences in their presence between step types

Muscle synergy analysis yielded on average 3.8 ± 1.0 muscle synergies per participant for reactive stepping, 4.1 ± 0.6 for voluntary stepping, and 3.9 ± 0.7 for motor-simulated stepping (χ^2^(2) = 2.48, *p* = 0.290). Of these individual muscle synergies, three were consistently observed across participants and step types (W1-W3) (Fig. 3). In reactive stepping, a majority of subjects exhibited a fourth group synergy (W4) that was less consistently present in voluntary steps, yet the difference in presence between step types only bordered significance (*p* = 0.085). A fifth group synergy (W5) was observed with a similarly low prevalence across step types (4–7 out of 15 participants). The structure (i.e. relative contribution of the individual muscles) of the group synergies showed high similarities across step types (*r* = 0.87–1.00).

**Fig. 4:**
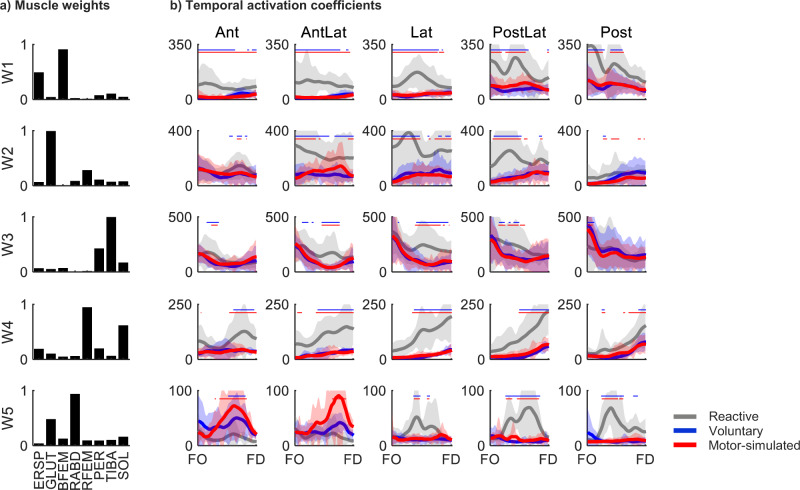
(a) Muscle weights averaged across step types. Each bar represents the contribution of that muscle. Erector spinae (ERSP), gluteus medius (GLUT), biceps femoris (BFEM), rectus abdominis (RABD), rectus femoris (RFEM), peroneus longus (PER), tibialis anterior (TIBA) and soleus (SOL). (b) Temporal activation coefficients, from the instants of foot off (FO) to foot down (FD), shown here as mean (thick solid lines) ± standard deviation (shaded area) for each of the step types per step direction. Significant differences between step types are indicated with blue (reactive vs. voluntary) and red (reactive vs. motor-simulated) horizontal lines

The time- and direction-dependent tuning of temporal activation coefficients differed between step types (Fig. 4). The temporal activation coefficients were rather similar between voluntary and motor-simulated stepping, without any significant differences between. In contrast, reactive stepping generally involved higher temporal activation coefficients than voluntary and motor-simulated stepping, with significant differences in many instances. The direction in which these differences were most pronounced varied between group synergies. For instance, in the case of muscle W2, W3 and W4, these differences were most pronounced in steps involving a sideward component (i.e., AntLat, Lat and PostLat)), whereas for group synergy W1 this was the case for steps with a posterior component.

### Step characteristics and body configurations

**Fig. 5:**
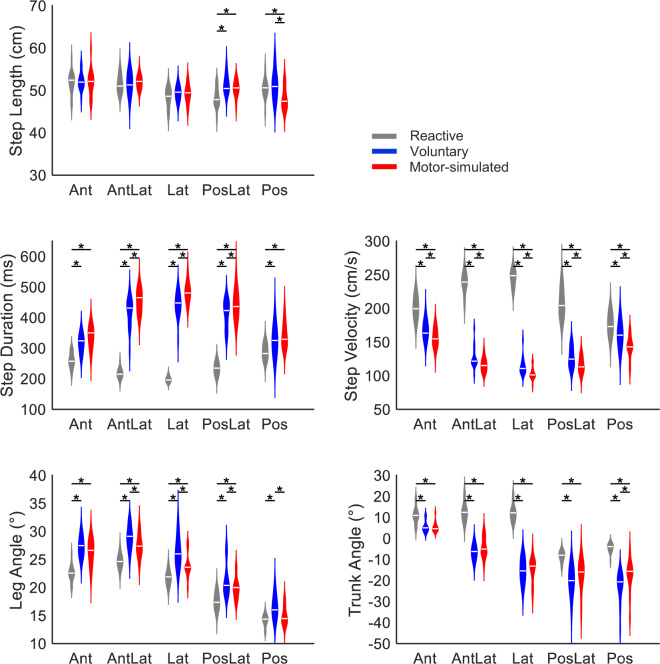
Violin plots of spatiotemporal step characteristics and body configurations. Larger positive leg angles correspond to a mid-pelvis position more distant from the foot position in the transverse plane. Larger positive trunk angles represent a greater trunk inclination towards the step target. The violin shapes represent the distribution of the data, the median is shown as white horizontal line. Asterisks (*) indicate significant differences between step types

For all step characteristics and body configuration outcomes, a significant interaction effect *of step type* with *step direction* was found (p-values < 0.01), indicating that the effect of step type differed between step directions. Post-hoc analysis for each step direction indicated comparable step lengths across step types, in agreement with the instructions given, albeit with minor significant differences in backward directed steps (Fig. 5). Step durations significantly differed between step types and were shortest during reactive stepping and longest during motor-simulated stepping. Differences in step durations corresponded with differences in step velocities, i.e., the velocity was higher with shorter step durations. In terms of body configuration (i.e., trunk and leg angles at stepping foot contact), leg angles were smaller whereas trunk angles were larger in reactive compared to voluntary steps in all directions. In (antero)lateral steps, trunk angles were positive in reactive steps (i.e. oriented in the direction of the step), whereas negative angles were observed in voluntary steps (i.e. trunk moving in the opposite direction). The angles of motor-simulated steps were generally in between those of voluntary and reactive stepping.

## Discussion

The primary aim of this study was to investigate the neuromuscular control of three different types of stepping. Our results show that the muscle weights were largely consistent between the step types. The temporal activation coefficients substantially differed, as well as the body configurations at the instant of foot down.

### Spatial muscle contributions

The novelty of our study lies in the comprehensive examination of muscle synergies of three different step types in multiple directions. Importantly, our experimental setup was successful in keeping step length reasonably constant across step directions and step types, which allowed us to fairly compare synergy recruitment between step types. In line with findings from previous studies that showed largely preserved muscle synergy structures during perturbed gait (Brüll et al. 2024; Chvatal and Ting 2012; Oliveira et al. 2012), it appears that a ‘core set’ of three group synergies was engaged regardless of step type from quiet stance. This indicates that step type does not profoundly influence the coactivation patterns of groups of muscles with their respective weightings. The muscle synergies in this core set closely resemble muscle synergies consistently observed in a previous study on reactive stepping following multidirectional balance perturbations in young and older adults (Staring et al. 2024). For reactive stepping, we identified a fourth group synergy (W4) in addition to the ‘core set’ of group synergies. This fourth group synergy was fairly consistently present in 67% of the participants, compared to 27% during voluntary stepping. This group synergy mainly consisted of rectus femoris and soleus activity, while these muscles had rather small contributions to the other group synergies. This indicates that concerted recruitment of these muscles may be essential for successfully recovering from a balance perturbation with a reactive step (van Lith 2020). Follow-up analyses of the VAF per step direction showed that this was most pronounced in the forward direction, where W4 contributed to 21.1% of the total VAF (compared to 6–12% in the other directions). Group synergy W4 matches another muscle synergy described by Staring et al. (2024). In line with our hypothesis, this group synergy was present in 47% of motor-simulated stepping trials, which is in between voluntary and reactive stepping, suggesting that at least some of the participants may have successfully recreated the muscle coactivation patterns of reactive stepping.

In line with this finding of intermediate W4 expression during motor-simulated stepping, the notable variability in leg and trunk angles among participants in this stepping condition indicated that a proportion of participants indeed attempted to adopt body configurations consistent with reactive stepping, whereas others did not (successfully). For instance, the participant in Fig. 1 demonstrated trunk inclination angles in the same direction during both reactive and simulated stepping, whereas the trunk was tilted in the opposite direction during voluntary stepping. Yet, in general, trunk angles during simulated stepping were closer to those of voluntary stepping.

The large variability in body configurations suggests that performance of the motor simulation task varied greatly between participants. This is in line with previous findings that motor imaging ability can vary significantly between individuals (McAvinue and Robertson 2008). Additionally, the use of the step mat in combination with our instructions to step along with the actor as accurately as possible may have put too much focus on simulating the actor’s foot placement. Instead, focusing the instruction on mental imagery of balance loss and simulation of the actor’s *whole-body* movements might result in greater similarities in neuromuscular recruitment between reactive and motor-simulated steps.

### Temporal activation coefficients

While differences in the muscle weights were modest, step type did substantially influence the temporal activation coefficients. Reactive stepping generally resulted in higher temporal activation coefficients, presumably due to the greater biomechanical challenge involved. In contrast to the self-initiated movement of the center of mass during voluntary stepping, a reactive step in response to a platform translation involves arresting the movement of the center of mass within the base of support (Shulman et al. 2018). Consequently, reactive stepping requires faster steps and therefore stronger muscle activation. The directions in which reactive stepping exhibited higher temporal activation coefficients varied between group synergies. The differences in temporal activation coefficients for group synergies W2-W4 were most pronounced for reactive steps with a lateral component (AntLat, Lat and PostLat). The greater temporal activation coefficients in these directions can at least partly be explained by the sideward platform translations that passively transfer the weight onto the (prospective) stepping leg (Gray et al. 2017). As a result, the stepping leg needs to be actively unloaded first, which leaves less time for completing the reactive step itself and thus requires an even faster and stronger activation of the muscles involved. Indeed, reactive step duration was shortest, especially in the directions with a lateral component, with particularly strong activation of the group synergy that included the hip abductors (W2).

### Strengths and limitations

Our focus was on the execution of the step itself, as voluntary and reactive step initiation are inherently different. Consequently, the observed muscle synergies cannot be compared one-to-one with those reported in other studies (Staring et al. 2024; Torres-Oviedo and Ting 2007; Wang et al. 2021). Moreover, different standardization procedures (e.g., whether or not amplitude normalization or normalization to unit variance is applied before extracting muscle synergies) could lead to different results. Furthermore, we recorded activity from eight leg and trunk muscles, with a relatively low number of muscles predominantly acting in the frontal plane. As a result, some differences in muscle coordination patterns may therefore have gone unnoticed for laterally-directed steps. Lastly, it is difficult to ascertain whether participants truly engaged with the motor simulation task as instructed (Wright et al. 2022), and if so, whether they imagined the kinesthetic sensations associated with postural changes following balance perturbations, or merely used the actor’s step as a cue for step direction instead.

### Conclusion and future directions

This study investigated the neuromuscular control of different types of stepping. Previous research already demonstrated that initiation of a voluntary and reactive step, prior to the actual step itself, inherently differs through the recruitment of anticipatory postural adaptations and automatic postural responses, respectively (Santos et al. 2010). In the present study, we showed that the neuromuscular control differs between voluntary and reactive stepping during the execution of the actual step as well. While we expected simulated stepping to be intermediate, its neuromuscular control generally resembled that of voluntary stepping. It requires further investigation whether simulated stepping does have benefits for improving reactive stepping performance. Additionally, it could be further investigated how the motor simulation task can be optimized, for example, by providing prior experience with balance perturbations, adding tactile stimuli, or emphasizing mental imagery of balance loss and simulation of *whole-body* movements. The findings could help enhance perturbation-based training interventions and could contribute to the development of effective home-based alternatives.

## Data Availability

Data will be made available by the corresponding author upon reasonable request.
